# Myeloid-derived suppressor cells in pleural effusion as a diagnostic marker for early discrimination of pulmonary tuberculosis from pneumonia

**DOI:** 10.3389/fimmu.2024.1390327

**Published:** 2024-04-29

**Authors:** Eun Sun Kim, Jahirul Islam, Hee-Jae Lee, Seung-Yong Seong, Je-In Youn, Byoung Soo Kwon, Se Joong Kim, Jae-Ho Lee

**Affiliations:** ^1^ Department of Internal Medicine, Seoul National University Bundang Hospital, Seoul National University College of Medicine, Seongnam, Republic of Korea; ^2^ Hospital Medicine Center, Seoul National University Bundang Hospital, Seongnam, Republic of Korea; ^3^ Wide River Institute of Immunology, Seoul National University College of Medicine, Hongcheon, Republic of Korea; ^4^ Department of Biomedical Sciences, Seoul National University College of Medicine, Seoul, Republic of Korea; ^5^ Department of Microbiology and Immunology, Seoul National University College of Medicine, Seoul, Shaperon Inc., Republic of Korea; ^6^ Department of Biochemistry, College of Life Science & Biotechnology, Yonsei University, Seoul, Republic of Korea; ^7^ SG Medical, 3-11, Ogeum-ro 13-gil, Songpa-gu, Seoul, Republic of Korea; ^8^ Division of Pulmonary and Critical Care Medicine, Seoul National University Bundang Hospital, Seongnam, Republic of Korea; ^9^ Division of Pulmonary and Critical Care Medicine, Department of Internal Medicine, Sheikh Khalifa Specialty Hospital, Ras Al Khaimah, United Arab Emirates

**Keywords:** myeloid-derived suppressor cells, pleural effusion, parapneumonic effusion, pneumonia, tuberculosis, tuberculous pleural effusion

## Abstract

**Introduction:**

Tuberculous pleural effusion (TPE) stands as one of the primary forms of extrapulmonary tuberculosis (TB) and frequently manifests in regions with a high prevalence of TB, consequently being a notable cause of pleural effusion in such areas. However, the differentiation between TPE and parapneumonic pleural effusion (PPE) presents diagnostic complexities. This study aimed to evaluate the potential of myeloid-derived suppressor cells (MDSCs) in the pleural fluid as a potential diagnostic marker for distinguishing between TPE and PPE.

**Methods:**

Adult patients, aged 18 years or older, who presented to the emergency room of a tertiary referral hospital and received a first-time diagnosis of pleural effusion, were prospectively enrolled in the study. Various immune cell populations, including T cells, B cells, natural killer (NK) cells, and MDSCs, were analyzed in both pleural fluid and peripheral blood samples.

**Results:**

In pleural fluid, the frequency of lymphocytes, including T, B, and NK cells, was notably higher in TPE compared to PPE. Conversely, the frequency of polymorphonuclear (PMN)-MDSCs was significantly higher in PPE. Notably, compared to traditional markers such as the neutrophil-to-lymphocyte ratio and adenosine deaminase level, the frequency of PMN-MDSCs emerged as a more effective discriminator between PPE and TPE. PMN-MDSCs demonstrated superior positive and negative predictive values and exhibited a higher area under the curve in the receiver operating characteristic curve analysis. PMN-MDSCs in pleural effusion increased the levels of reactive oxygen species and suppressed the production of interferon-gamma from T cells following nonspecific stimulation. These findings suggest that MDSC-mediated immune suppression may contribute to the pathology of both TPE and PPE.

**Discussion:**

The frequency of PMN-MDSCs in pleural fluid is a clinically useful indicator for distinguishing between TPE and PPE.

## 1 Introduction

Tuberculous pleural effusion (TPE) is the second most common manifestation of extrapulmonary tuberculosis (TB) after TB lymphadenitis, accounts for 15–25% of TB cases globally and represents a frequent origin of pleural effusion in TB-endemic regions (1–6). TPE originates from *Mycobacterium tuberculosis* (MTB) infection in the pleura, inducing pronounced and chronic inflammation characterized by fluid and inflammatory cell accumulation in the pleural space (2). The gold standard for TPE diagnosis relies on identifying MTB in either pleural fluid or biopsy samples (2, 7–9). However, direct examinations of pleural fluid for acid-fast bacilli typically yield negative results in immunocompetent individuals, unless the patient has a tuberculous empyema (10, 11). Culture study for MTB also has limitations of low sensitivity and the extended 8-week period required to definitively rule out the infection, thereby causing delays in making crucial clinical decisions (7–9). Additionally, the invasive nature of pleural biopsies poses a relatively high risk of complications (9).

Parapneumonic pleural effusion (PPE) is a commone complication of pneumonia, comprising approximately 2-3% of all pneumonia cases, with recent studies suggesting an increasing prevalence (12–15). Diagnosis is typically based on clinical signs, symptoms, pleural fluid analysis, characterized by neutrophilic exudate, and microbiologic examination of the pleural fluid with appropriate stains and cultures (12–16). Definitive diagnosis of complicated cases may require pleural biopsy, often via thoracoscopy, to identify microorganisms in affected tissue (16, 17). Clinically, distinguishing between TPE and PPE is challenging due to overlapping symptoms like cough, chest pain, and fever (18).

The presumptive diagnosis of TPE has traditionally relied on the predominance of lymphocytes and a high concentration of adenosine deaminase (ADA) in pleural effusion (2). The differentiation between TPE and PPE is typically determined through pleural fluid analysis. This involves identifying a lymphocyte-dominated exudate with ADA levels exceeding 40 U/L for TPE, contrasting with a neutrophil-dominated exudate for PPE (3). However, recent research has revealed an increased occurrence of neutrophil-dominant TPE, challenging this traditional classification (19, 20). Elevated ADA levels have been observed in samples of pleural effusion from varying causes: about two-thirds of cases of empyema and approximately one-third of cases of PPE are characterized by ADA levels surpassing 40 U/L (2). Hence, depending solely on elevated ADA levels in lymphocyte-dominated effusions for diagnosis poses the risk of misclassifying TPE as PPE. Such misdiagnosis could delay appropriate treatment for TPE, potentially exacerbating the patient’s clinical condition. Although additional pleural fluid assays and biomarkers, including B-cell response, complement activation and various cytokines (21, 22), for the diagnosis of TPE have been explored, none of these have gained endorsement for TPE diagnosis owing to their limited sensitivity and/or specificity (23).

The pathogenesis of TB disease is closely linked to the suppression of cell-mediated immunity, with myeloid-derived suppressor cells (MDSCs) playing a prominent role as immunosuppressive cells (24). It is well-documented that MDSCs increase in number during the progression of TB (25, 26). Myeloid-derived suppressor cells (MDSCs) constitute a diverse and immunosuppressive cell population. This population is generally characterized by its heterogeneity, with two main types: polymorphonuclear-MDSC (PMN-MDSC), represented by the phenotype CD14^-^CD11b^+^CD33^+^CD15^+^; and monocytic-MDSC (M-MDSC), represented by CD11b^+^HLA-DR^-^CD14^+^. A recent study has demonstrated that the frequency of PMN-MDSCs could serve as a distinguishing factor between latent TB and active TB (27). Evidence indicates an increase in regulatory T cell, natural killer (NK) cells, and MDSCs in the transition from blood circulation to the disease site in TB patients (25, 28, 29). Additionally, the immune composition in the pleural fluid of patients with TPE differs significantly from that in the peripheral blood (30). However, research examining MDSCs in pleural effusion samples, as well as the role of MDSCs in the differential diagnosis of pleural effusion, is scarce.

Accordingly, our hypothesis suggests that MDSCs could serve as a means to distinguish between TPE and PPE, each demanding tailored treatment strategies due to the challenge in precise classification based solely on clinical criteria. Thus, this study aimed to evaluate the potential of MDSCs in pleural fluid as a diagnostic marker for distinguishing between TPE and PPE. If the usefulness of the MDSC-based diagnostic approach is confirmed through this study, rapid and accurate diagnosis of TPE and PPE will be possible. These advances are expected to facilitate prompt treatment decisions and potentially improve patient outcomes. Toward this goal, we examined matched blood and pleural fluid samples obtained from patients diagnosed with pleural effusion, later categorized as either TPE or PPE. The immune cell frequency of PMN-MDSCs was analyzed using flow cytometry to evaluate their potential as a superior, reliable, and accurate diagnostic marker for the early discrimination of TPE from PPE.

## 2 Methods

### 2.1 Study design and participants

This prospective cohort study was approved by the institutional review board of Seoul National University Bundang Hospital (IRB No. B-1512-328-302) and was conducted according to the tenets of the Declaration of Helsinki. All patients provided informed consent prior to study participation.

We enrolled consecutive adult patients (aged ≥18 years) diagnosed with pleural effusion upon their visit to the emergency department at the Seoul National University Bundang Hospital, a tertiary referral hospital in Seoul Korea, between March 2016 and February 2018. The patients who underwent thoracentesis for diagnostic or therapeutic purposes and provided written consent to participate were included in this study. The exclusion criteria were: patients who received antibiotics or anti-TB medications within the 24 h preceding thoracentesis; those receiving immunosuppressants for rheumatic diseases; those with immunological deficiencies such as acquired immunodeficiency syndrome; those having hematologic malignancies such as leukemia; and pregnant or breastfeeding individuals. Patients who had received leukocyte growth factor prior to the measurement of immunosuppressive cells were also excluded.

### 2.2 Definitions

Pleural TB diagnosis relied on the following criteria (1): Positive results on acid-fast bacilli smear, TB culture, or TB polymerase chain reaction (PCR) in the pleural fluid sample (2).; histopathological findings from pleural biopsy revealing granuloma without an apparent cause for granulomatous lung disease; (3) positive sputum culture for TB, followed by resolution of the pleural effusion after empirical TB treatment; or (4) a lymphocyte-dominated exudate lacking malignant cells, with ADA levels surpassing 40 IU/L, demonstrating improvement during TB treatment (20). PPE was defined as being associated with a focal lung infection, such as bacterial pneumonia, lung abscess, or infected bronchiectasis (31). The diagnosis necessitated the microbiological identification of a pathogenic organism in pleural effusion or improvement observed through antibiotic treatment and/or thoracotomy, without TB or cancer cells in the pleural fluid (31, 32). Lymphocyte or neutrophil predominance was identified when the proportion of lymphocytes or neutrophils exceeded 50% of the total leukocyte count in the fluid. Basophils, monocytes, and eosinophils were collectively categorized as “other” cell types.

### 2.3 Analysis of immune cells in peripheral blood and pleural fluid samples

Blood samples were individually processed within 6 hours from collection in the hospital setting to ensure minimal degradation and accurate representation of the immunophenotyping profiles. Peripheral blood mononuclear cells (PBMCs) were isolated from whole blood using Ficoll (GE Healthcare; Chicago, IL, USA) density gradient centrifugation, and single-cell populations were prepared by centrifugation of pleural fluid from both TPE and PPE patients. PBMCs and pleural effusion cells were washed in Dulbecco’s phosphate-buffered saline and suspended in FACS buffer (Dulbecco’s phosphate-buffered saline containing 1% fetal bovine serum with 1 mM EDTA). The following anti-human antibodies were used: anti-CD45 (clone HI30), anti-CD3 (clone UCHT1), anti-CD4 (clone L200), anti-CD8 (clone SK1), anti-CD19 (clone SJ25C1), anti-CD56 (clone B159), anti-CD25 (clone M-A251), anti-CD15 (clone HI98), anti-CD14 (clone MQP9), anti-HLA-DR (clone G46-6), anti-CD33 (clone P67.6), anti-CD11b (clone 1CRF44) purchased from BD Biosciences, anti-CD127 (clone eBioRDR5, eBioscience) and anti-Foxp3 (clone 206D, Biolegend). PBMCs (1×10^6^) and pleural fluid cells were incubated with fluorochrome-conjugated monoclonal antibody for 15 min at 20~22°C. Samples were washed in Dulbecco’s phosphate-buffered saline, suspended in FACS buffer containing diaminophenylindole (0.3 µg/ml) and immediately acquired using BD LSRFortessa flow cytometer (BD Biosciences, Franklin Lakes, NJ, USA). Immune cell frequencies were analyzed using FlowJo software (FlowJo LLC, Ashland, OR, USA).

**Figure 1:**
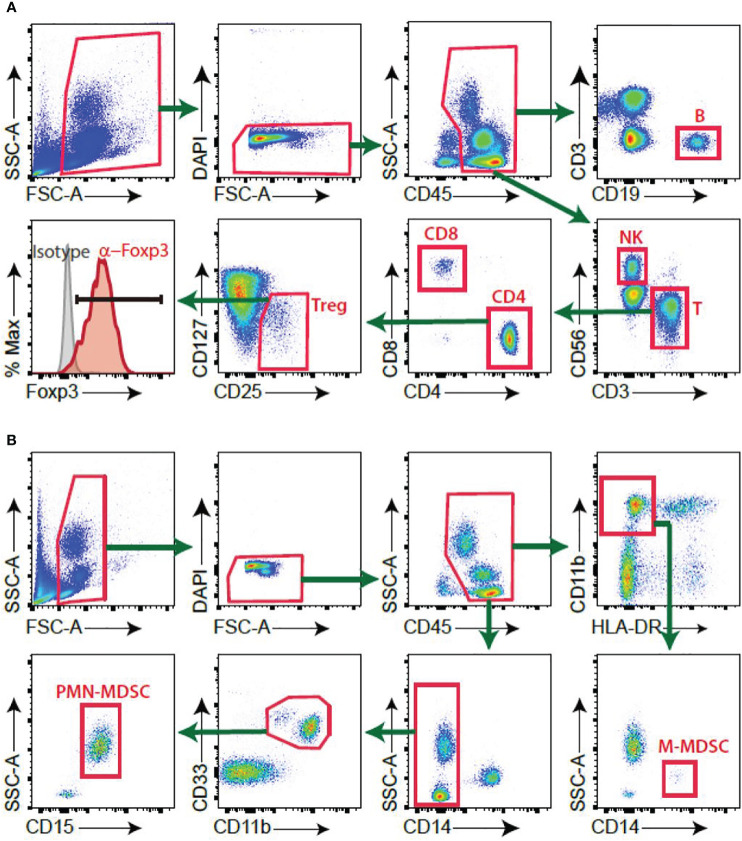
Gating strategy of immune cells. **(A)** B, NK, T, Treg cells **(B)** PMN-MDSC, M-MDSC.

Different human lymphocyte populations were measured in pleural fluid and PBMCs from patients with TPE and PPE using flow cytometry. Lymphocytes were gated from CD45^+^ leukocyte population to eliminate the exudative erythrocytes. B, T, NK, CD4^+^ T, CD8^+^ T and Treg cells were targeted among the lymphocytes (**Figure 1**).

### 2.4 Reactive oxygen species production

The oxidation-sensitive dye dichlorodihydrofluorescein diacetate (DCFDA) (C6827, Molecular Probes/Invitrogen, Carlsbad, CA, USA) was used to measure reactive oxygen species (ROS) production. Cells were labelled with surface markers, washed, and incubated with the serum-free Roswell Park Memorial Institute media in the presence of 3 μM dichlorodihydrofluorescein diacetate at 37°C. After a 30-min incubation, the cells were washed with phosphate-buffered saline and analyzed using flow cytometry.

### 2.5 Immune suppression functional assay

PBMCs containing 20–30% T cells which were measured by flow cytometry were cultured for 48 h in a human interferon gamma (IFN-γ) (3 µg/ml) (554699, BD) precoated plate (Millipore, Burlington, MA, USA) with or without different doses (1:1, 1:3, 1:9) of PMN-MDSC from pleural effusion cells. Culture conditions were optimized with granulocyte-macrophage colony-stimulating factor (10 ng/ml), interleukin-2 (10 ng/ml), CD3 (1.25 µg/ml) (555329; BD Biosciences), and CD28 (12.5 µg/ml) (555725; BD Biosciences). A negative control well with 200,000 effector PBMCs with granulocyte-macrophage colony-stimulating factor (10 ng/ml) and interleukin-2 (10 ng/ml) only without CD3/CD28 stimulation was prepared. Biotinylated anti-human-IFN-γ (554550; BD Biosciences), streptavidin-alkaline phosphatase conjugate (554065; BD Biosciences), and 5-bromo-4-chloro-3-indolyl phosphate/nitroblue tetrazolium chromogen were utilized for the spot development. The number of spot-forming cells was determined by subtracting the average number of spots observed in the negative control wells from the average number of spots observed in each stimulation condition.

### 2.6 Statistical analysis

Differences between the TPE and PPE groups were analyzed using unpaired t-tests. Receiver operating characteristic (ROC) curve analysis was used to identify the greatest sum of sensitivity and specificity for identifying superior diagnostic markers between the patient groups. An ROC curve is typically considered to indicate good diagnostic performance when the area under the curve (AUC) is ≥ 0.8 (33, 34). All statistical analyses were performed using GraphPad Prism (GraphPad Software, Boston, MA, USA). A p-value of <0.05 was considered statistically significant.

## 3 Results

### 3.1 Baseline patient characteristics

In total, 47 patients were enrolled; among them, 17, 14, 14, and 2 patients were diagnosed with PPE, TPE, malignant pleural effusion, and transudative effusion, respectively. The definitive diagnosis of TPE in 14 patients was established clinically through either a positive sputum culture for TB, coupled with resolution of the pleural effusion following empiric TB treatment, or by identifying a lymphocytic-predominant exudate with ADA levels surpassing 40 IU/L, demonstrating improvement during TB therapy. Conversely, the final diagnosis of PPE was based on pleural fluid analysis revealing an exudate devoid of TB or cancer cells, which showed improvement after an average of one week of antibiotic therapy and exhibited no recurrence. Blood and pleural fluid samples from patients with PPE and TPE were screened, with samples from the two transudative cases serving as controls. **Table 1** outlines the baseline characteristics of the enrolled patients. There were no significant differences in age and underlying comorbidities between patients with TPE and PPE. All patients with transudative pleural effusion exhibited congestive heart failure. Initial laboratory, radiological, and effusion profiles are summarized in **Table 2**. Laboratory tests showed elevated white blood cell counts, particularly neutrophils and C-reactive protein levels, in patients with PPE than in those with TPE. Examination of effusion profiles revealed lower levels of lactate dehydrogenase, higher lymphocyte fractions, and significantly elevated ADA levels in patients with TPE. Radiologically, no significant differences were observed between the two groups.

**Table 1:** Baseline patient characteristics.

	Total (n = 47)	PPE (n = 17)	TPE (n = 14)	MPE (n = 14)	Control (n = 2)	**p Value	*p Value	p Value
Male sex	35 (74.5)	15 (88.2)	9 (64.3)	10 (71.4)	1 (50.0)	0.120	0.291	0.368
Age, years	69.0 (18–91)	69.0 (21–91)	70.0 (18–83)	63.5 (46–82)	78.0 (71–85)	0.592	0.771	0.587
BMI, kg/m^2^	22.2 (8.0)	22.2 (4.5)	20.2 (2.7)	24.9 (13.4)	17.9 (2.7)	0.156	0.325	0.408
Ever-smoker	22 (46.8)	9 (52.9)	7 (50.0)	5 (35.7)	1 (50.0)	0.876	0.622	0.798
Smoking, PY	0.0 (0–50)	1.3 (0–50)	0.5 (0–40)	0.0 (0–45)	10.0 (0–20)	0.169	0.879	0.962
Comorbidities								
DM	13 (27.7)	4 (23.5)	2 (14.3)	6 (42.9)	1 (50.0)	0.533	0.228	0.637
HTN	20 (42.6)	7 (41.2)	5 (35.7)	7 (50.0)	1 (50.0)	0.766	0.755	0.885
Previous TB	6 (12.8)	3 (17.6)	2 (14.3)	0 (0.0)	1 (50.0)	0.808	0.283	0.739
CLD	4 (8.5)	3 (17.6)	1 (7.1)	0 (0.0)	0 (0.0)	0.402	0.231	0.260
CKD	6 (12.8)	3 (17.6)	1 (7.1)	2 (14.3)	0 (0.0)	0.402	0.703	0.383
CHF	6 (12.8)	3 (17.6)	1 (7.1)	0 (0.0)	2 (100.0)	0.402	0.231	0.001
CVD	3 (6.4)	2 (11.8)	0 (0.0)	1 (7.1)	0 (0.0)	0.197	0.442	0.183
Previous malignancy	14 (29.8)	3 (17.6)	2 (14.3)	8 (57.1)	1 (50.0)	0.808	0.017	0.042

**p-value: PPE vs TPE, *p-value: PPE vs TPE vs MPE, p-value: PPE vs TPE vs MPE vs control.

BMI, body mass index; PY, pack-years; CHF, chronic heart failure; CKD, chronic kidney disease; CLD, chronic liver disease; CVD, coronary vascular disease; DM, diabetes mellitus; HTN, hypertension; MPE, malignant pleural effusion; PPE, parapneumonic effusion; TB, tuberculosis; TPE, tuberculous pleural effusion.

**Table 2:** Initial laboratory, radiologic, and effusion chemistry profiles.

	Total (n = 47)	PPE (n = 17)	TPE (n = 14)	MPE (n = 14)	Control (n = 2)	**p Value	*p Value	p Value
Laboratory findings								
WBC, cells/mm^3^	11.5 (7.4)	16.5 (9.6)	7.9 (3.6)	9.4 (3.9)	9.9 (5.1)	0.003	0.001	0.004
Neutrophils, %	77.7 (10.1)	83.5 (9.3)	73.9 (10.8)	73.9 (6.4)	82.8 (17.0)	0.013	0.005	0.014
Lymphocytes, %	12.6 (7.6)	7.8 (5.4)	15.2 (7.8)	15.9 (6.9)	11.7 (11.7)	0.004	0.002	0.007
CRP, mg/dL	10.3 (9.1)	19.2 (7.7)	7.8 (5.7)	3.2 (2.8)	1.9 (2.3)	<0.001	<0.001	<0.001
Procalcitonin, ng/mL	1.8 (4.8)	2.7 (6.0)	0.2 (0.2)	0.4 (0.3)	0.3 (0.0)	0.425	0.643	0.801
Radiologic findings								
Loculated effusion	20 (42.6)	11 (64.7)	8 (57.1)	1 (7.1)	0 (0.0)	0.679	0.002	0.004
Consolidations	20 (42.6)	12 (70.6)	7 (50.0)	1 (7.1)	0 (0.0)	0.256	0.001	0.002
Effusion profiles								
pH	7.5 (0.2)	7.3 (0.2)	7.5 (0.2)	7.6 (0.1)	7.8 (0.0)	0.113	0.102	0.101
Protein, mg/dL	4.2 (1.0–5.0)	4.2 (1.0–5.0)	4.2 (1.3–5.0)	4.3 (3.0–4.9)	1.5 (1.2–1.9)	0.753	0.524	0.003
Glucose, mg/dL	92 (1–425)	43 (1–425)	88 (1–119)	133 (50–325)	122 (103–141)	0.972	0.078	0.141
LDH, U/L	488 (50–4000)	1076 (50–4000)	365 (177–1309)	355 (71–3707)	59 (58–60)	0.002	0.005	0.006
RBC, ×10^2^, cells/mm^3^	28.0 (0.5–7130)	30.0 (2–120)	16.5 (80–28000)	215.0 (4–7130)	0.55 (0.5–0.6)	0.309	0.006	0.013
WBC, ×10^2^, cells/mm^3^	12.0 (0–1024)	28.4(11.2–1024)	10.4 (0–102)	6.4 (1.3–32)	4.5 (3.0–6.1)	0.135	0.074	0.132
Neutrophils, %	32.9 (36.2)	69.3 (28.1)	12.9 (24.2)	15.1 (22.9)	5.0 (1.4)	<0.001	<0.001	<0.001
Lymphocytes, %	34.3 (31.5)	7.8 (8.6)	58.9 (34.6)	36.4 (23.2)	58.5 (5.0)	<0.001	<0.001	<0.001
Others, %	28.5 (22.6)	22.9 (21.3)	13.9 (7.9)	48.4 (21.9)	36.5 (3.5)	0.016	<0.001	<0.001
ADA, IU/L	33.0 (3.0–171.0)	35.5 (4.0–171.0)	80 (23.0–139.0)	21.0 (11.0–39.0)	5.0 (3.0–7.0)	0.016	<0.001	<0.001
CEA, ng/mL	1.4 (1–2963)	1.1 (1.0–5.9)	1.3 (1.0–2.6)	7.9 (1.0–2963.0)	1.1 (1.1)	0.567	0.315	0.499

**p-value: PPE vs TPE, *p-value: PPE vs TPE vs MPE, p-value: PPE vs TPE vs MPE vs control.

ADA, adenosine deaminase; CEA, carcinoembryonic antigen; CRP, c-reactive protein; LDH, lactate dehydrogenase; MPE, malignant pleural effusion; PPE, parapneumonic effusion; RBC, red blood cell; TPE, tuberculous pleural effusion; WBC, white blood cell.

### 3.2 Patients with TB had Significantly higher lymphocyte counts in pleural fluid than those with PPE

**Figure 2:**
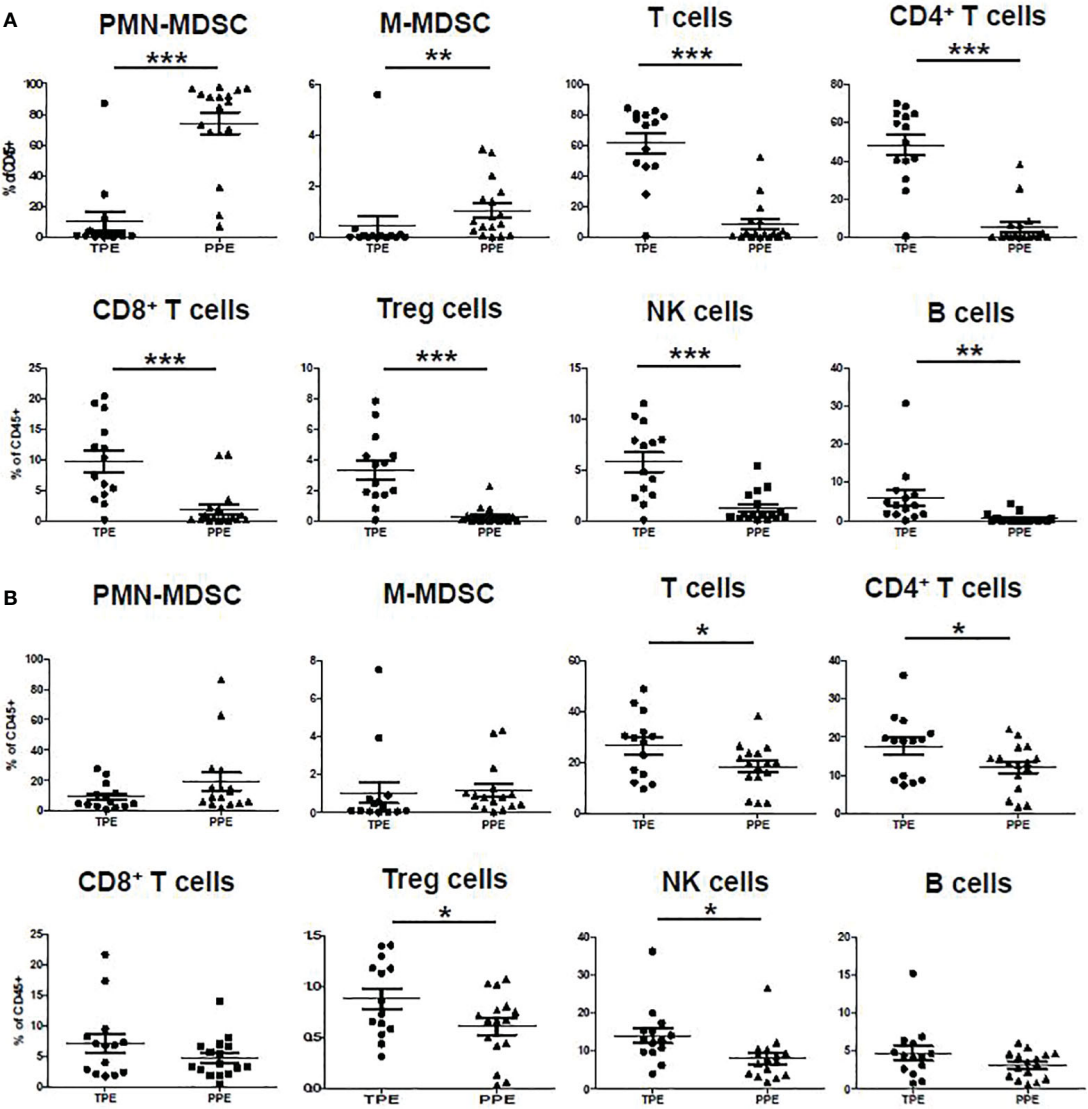
Immune cell frequency in **(A)** pleural effusion and **(B)** blood of TPE and PPE. *p < 0.05, **p < 0.01, ***p < 0.001.

The measured lymphocyte populations (T, CD4^+^ and CD8^+^ T, NK, and Treg cells) in the pleural effusion fluid showed higher frequencies in the TPE group than in the PPE group (**Figure 2A**). Similarly, PBMCs from TB patients also showed higher lymphocyte populations (T, CD4^+^, Treg, and NK cells) (**Figure 2B**).

### 3.3 Patients with PPE had significantly higher MDSC in pleural fluid than those with TB

**Figure 3:**
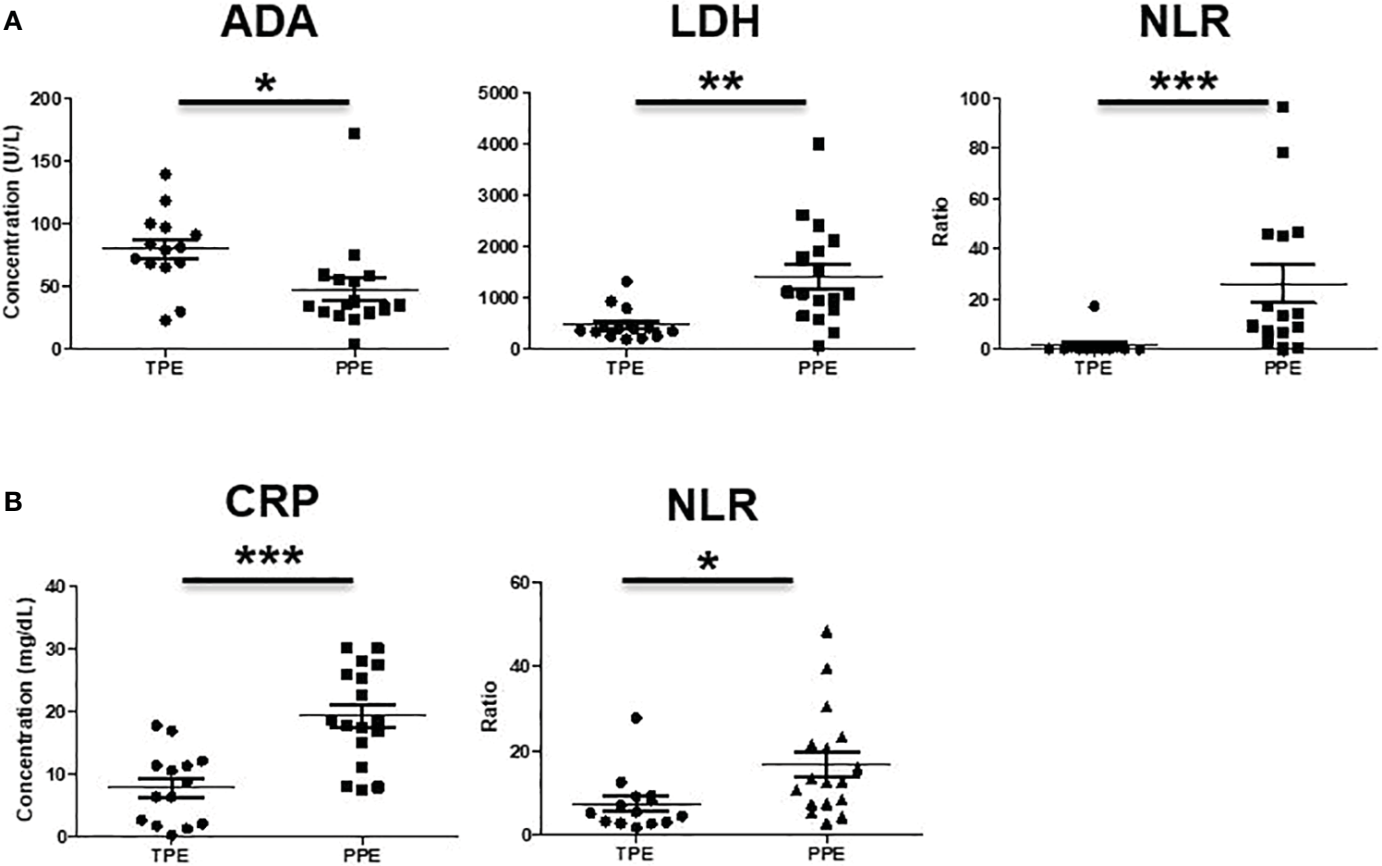
Analysis of **(A)** ADA, LDH, NLR in pleural effusion and **(B)** CRP, NLR in blood of TPE and PPE. *p < 0.05, **p < 0.01, ***p < 0.001.

In the CD45^+^ population, CD14^-^CD33^+^CD11b^+^CD15^+^ were considered PMN-MDSC and CD11b^+^HLA-DR^-^CD14^+^ were considered M-MDSC (**Figure 1**). The frequencies of PMN-MDSC and M-MDSC in pleural fluid were higher in patients with PPE than in those with TPE (**Figure 2A**). In PBMCs, PMN-MDSC and M-MDSC were not significantly increased (**Figure 2B**). Additionally, ADA was significantly lower in patients with PPE than in those with TPE, whereas lactate dehydrogenase (LDH) and neutrophil–lymphocyte ratio (NLR) were substantially higher in patients with PPE (**Figure 3A**). The blood levels of C-reactive protein (CRP) and NLR were significantly higher in patients with PPE than in those with TPE (**Figure 3B**).

### 3.4 Diagnostic accuracy for discriminating between TB and PPE

**Figure 4:**
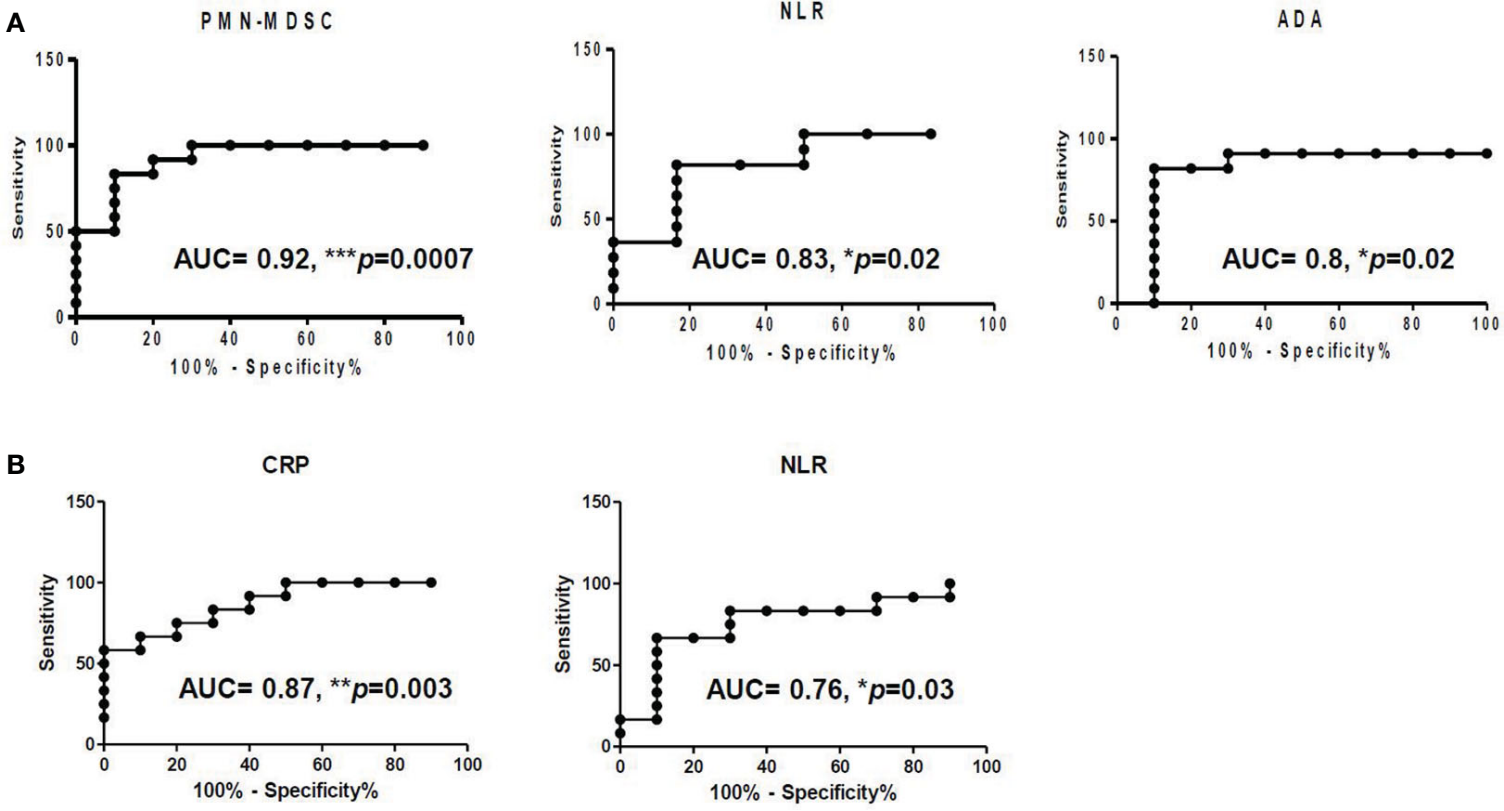
Receiver operating characteristic (ROC) of **(A)** PMN-MDSC, NLR, and ADA in pleural fluid and of **(B)** CRP and NLR in blood.

In pleural effusion, NLR (AUC: 0.83, p = 0.02), ADA (AUC: 0.8, p = 0.02), and C-reactive protein in blood (AUC: 0.87, p = 0.003) demonstrated robust diagnostic capabilities. However, compared to these biomarkers, PMN-MDSC in pleural fluid exhibited superior discriminatory power between TPE and PPE (AUC: 0.92, p = 0.0007) (**Figure 4**).

### 3.5 Characterization of PMN-MDSC in pleural effusion

**Figure 5:**
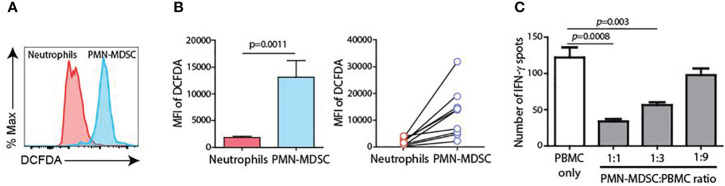
Charateristics of PMN-MDSC. **(A)** ROS production between blood neutrophils and PMN-MDSC from pleural fluid. **(B)** Inhibition of IFN-γ production of T cells by PMN-MDSC from pleural effusion. **(C)** Inhibition of IFN-γ production from T cells by PMN-MDSC from pleural effusion.

CD45^+^CD11b^+^CD15^+^ cells from pleural fluid (PMN-MDSC) and blood (neutrophils) were isolated, and major immune suppression factor of PMN-MDSC (reactive oxygen species, ROS) was measured. ROS production was significantly higher in PMN-MDSC from pleural fluid than in blood neutrophils (**Figure 5A**). In addition, PMN-MDSC from pleural effusion cells suppressed IFN-γ production from T cells in a dose-dependent manner, suggesting that the pleural effusion comprised immune-suppressive PMN-MDSCs (**Figure 5B**).

## 4 Discussion

TPE is strongly considered in patients presenting with lymphocytic exudative pleural effusion and an elevated ADA value >40 U/L (23). However, TPE can manifest in various forms, including simple exudative, suppurative, chylous, or pseudochylous presentations. Initially characterized by a preponderance of PMN leukocytes, the effusion undergoes a subsequent transition marked by an influx of macrophages. This phase is followed by a spectrum of lymphocyte-dominant exudation (23). Given this variability, distinguishing TPE from PPE based solely on conventional biochemical analyses or ADA values is challenging (9). Moreover, for patients presenting with conditions causing transudative effusions, such as congestive heart failure or liver cirrhosis, TPE itself may not exhibit characteristics typical of an exudate. The correlation between MDSCs and the severity of TB suggests a potential mechanism by which MDSCs contribute to TB progression through immunosuppression (35). Previous studies have demonstrated an increase in MDSCs in the blood of patients with TB, which is associated with a weakened immune response against MTB (36–39). However, the specific role of MDSCs in the pleural fluid of patients with TPE or PPE remains unclear.

This study was performed with the understanding that investigating the presence and role of MDSCs in the pleural fluid of these patients would yield valuable insights into the immunopathogenesis of pleural TB and bacterial pneumonia (25–27, 30). Our results support a promising diagnostic role for the frequency of immune cells in pleural fluid, assessed through flow cytometry, as a potential marker for distinguishing TPE from PPE. The effusion profile in this study showed that patients with TPE had a higher lymphocyte fraction and significantly higher ADA levels than those with PPE, consistent with previous findings (3). The results indicated that compared to conventional biomarkers such as NLR and ADA, PMN-MDSC exhibited superior discriminatory power in distinguishing between TPE and PPE (**Figure 4**). This heightened discriminative ability of PMN-MDSC may be attributed to several factors. First, PMN-MDSCs are a specialized subset of MDSCs that exert potent immunosuppressive effects, particularly in the context of chronic inflammatory conditions such as TB. The accumulation of PMN-MDSCs within the pleural space during TPE could reflect a distinct immunological response to MTB infection. This leads to alterations in the cellular microenvironment that are not fully captured by conventional inflammatory markers such as NLR and ADA.

Additionally, PMN-MDSCs may modulate the local immune response in a manner that facilitates the evasion of host defense mechanisms by MTB. This exacerbates disease progression and contributes to the observed differences in discriminatory ability between TPE and PPE. Furthermore, the distinct immunosuppressive characteristics of PMN-MDSCs, including their capacity to inhibit T cell proliferation and cytokine production, have significant implications for the immunopathogenesis of TPE and the clinical outcomes in patients with TPE and PPE (35, 40–42).

In our study, PMN-MDSCs isolated from pleural fluid exhibited high levels of ROS production and suppressed IFN-γ production in T cells upon nonspecific stimulation (**Figure 5**). These findings suggest that MDSC-mediated immunosuppression may play a role in the development and progression of TPE, potentially impacting its pathology and clinical manifestations. Future studies elucidating the mechanistic underpinnings of PMN-MDSC-mediated immunosuppression in TPE are warranted to validate their utility as a novel biomarker and explore their therapeutic potential in mitigating disease severity and improving patient outcomes.

This study has the advantage of prospectively enrolling patients presenting with pleural effusion during their initial hospital visit, where the etiology was unknown. Pleural fluid and blood samples were promptly collected and analyzed. Furthermore, exposure to antibiotics or anti-TB drugs, which could potentially influence immune cells, was strictly restricted to within a 24-hour window. Nevertheless, a limitation of this study lies in the stringent patient inclusion criteria, resulting in the exclusion of numerous patients from enrollment. Additional research targeting a larger cohort of patients is imperative for a more comprehensive elucidation of the study findings.

In conclusion, this study sheds light on the pivotal role of MDSCs in distinguishing between TPE and PPE. By analyzing paired blood and pleural fluid samples, we demonstrated significant differences in the immune profiles between TPE and PPE, with PMN-MDSC frequency emerging as a superior discriminator. The findings highlight the potential of PMN-MDSCs as novel biomarkers for improving the accuracy of differential diagnosis in pleural effusions. Moreover, the observed immunosuppressive properties of PMN-MDSCs suggest their involvement in the immunopathogenesis of both TPE and PPE. These insights have important clinical implications, offering opportunities for the development of targeted diagnostic and therapeutic strategies to enhance patient management and outcomes in pleural effusion cases.

## Data availability statement

The original contributions presented in the study are included in the article/supplementary material. Further inquiries can be directed to the corresponding author.

## Ethics statement

The studies involving humans were approved by Seoul National University Bundang Hospital (IRB No. B-1512-328-302). The studies were conducted in accordance with the local legislation and institutional requirements. The participants provided their written informed consent to participate in this study.

## Author contributions

EK: Conceptualization, Data curation, Formal analysis, Investigation, Project administration, Resources, Writing – original draft, Methodology. JI: Data curation, Formal analysis, Methodology, Writing – original draft, Conceptualization, Investigation. HL: Formal analysis, Investigation, Supervision, Writing – review & editing. SS: Data curation, Formal analysis, Investigation, Supervision, Writing – review & editing. JY: Conceptualization, Formal analysis, Funding acquisition, Investigation, Methodology, Project administration, Resources, Software, Supervision, Writing – review & editing. BK: Investigation, Resources, Supervision, Writing – review & editing. SK: Conceptualization, Methodology, Writing – review & editing. JL: Conceptualization, Funding acquisition, Investigation, Resources, Supervision, Writing – review & editing.
